# Contexts and Outcomes of Proxy Online Health Information Seeking: Mixed Studies Review With Framework Synthesis

**DOI:** 10.2196/34345

**Published:** 2022-06-24

**Authors:** Reem El Sherif, Pierre Pluye, Fidelia Ibekwe

**Affiliations:** 1 Department of Family Medicine McGill University Montreal, QC Canada; 2 School of Journalism & Communication Aix-Marseille University Aix-en-Provence France

**Keywords:** online health information, information seeking behavior, proxy information seeking, surrogate seekers, information outcomes, social support, health information, online information

## Abstract

**Background:**

High-quality online health information (OHI) can reduce unnecessary visits to health professionals and improve health. One of the ways that people use OHI is to support others with health conditions through proxy OHI seeking. Members of a person’s social circle may help them overcome information-seeking barriers and illness challenges. There are several models on proxy information seeking. Yet, we know little about the use and outcomes of OHI on behalf of someone else.

**Objective:**

The objectives of this paper are to explore and revise a framework on the context and outcomes of proxy OHI seeking

**Methods:**

We conducted a mixed studies literature review integrating qualitative and quantitative evidence with thematic analysis of the findings of 28 studies, followed by framework synthesis incorporating the derived themes.

**Results:**

We explored 4 main themes: (1) characteristics of proxy seekers, (2) context of proxy OHI seeking, (3) use of OHI to provide social support, and (4) outcomes of proxy OHI seeking. Our conceptual framework incorporates these themes and builds on previous work.

**Conclusions:**

By better understanding how people use information together, information providers can adapt the information to meet all users’ needs.

## Introduction

### Background

Two-thirds (67%) of respondents to the American Health Information National Trends Survey between 2008 and 2017 reported turning to the internet first for health information [1]. Similarly, 69% of Canadians reported using the internet to search for health information in 2020 [2], and the proportion of adults seeking online health information (OHI) in other Organisation for Economic Cooperation and Development (OECD) countries more than doubled between 2008 and 2017 [3]. The use of OHI can improve quality of life and is generally associated with positive outcomes, such as increased empowerment of seekers and their families and improved health outcomes [4-7].

Based on the results of a recent systematic review on the outcomes of OHI seeking (hereafter, OHI outcomes), several contextual factors associated with these outcomes were identified, such as age, education, income, and eHealth literacy [8]. Another contextual factor is social support, defined broadly as “support accessible to an individual through social ties to other individuals, groups, and the larger community”[9]. Social support is an important factor because one of the ways people use OHI is to support family members or friends with health conditions [10]. In fact, recent studies report that 61%-66% of OHI seekers are proxy seekers, meaning they seek OHI on behalf of someone else [11,12]. Moreover, findings from a study exploring internet use trends between 2008 and 2013 showed a significant increase in the use of family and friends to obtain health information [13].

However, while proxy information seeking has been explored in the literature, especially as it relates to health information, little is known about its relationship with the outcomes of OHI. This is a critical knowledge gap; previous research examining how to reduce negative outcomes of OHI suggests that OHI seekers may be able to overcome low eHealth literacy by discussing the information they find with others [14]. People are sometimes more likely to turn to their social circle to make sense of information they find rather than discuss it with a health professional [11,15]. Members of a person’s social circle may help them overcome information-seeking barriers and illness challenges (eg, if they are too physically weak or mentally incapacitated to search themselves) [15].

By better understanding how people and their social circles use information together, information providers can better adapt the information to meet both their needs, and public health interventions can target patients’ friends and family with information for dissemination and use [16]. Accordingly, the purpose of this paper is to contribute to our understanding of the role of social support in online health information outcomes by focusing on the outcomes of proxy OHI seeking.

This review will focus on the intersection of 3 main constructs: proxy information seeking, social support, and OHI outcomes.

#### Proxy Information Seeking

Information seeking encompasses “all the information that comes to a human being during a lifetime, not just in those moments when a person actively seeks information” [17]. In active information seeking mode, monitoring and directed searching are ways to answer known information needs (that are recognized and articulated). There are intervening variables that may be related to personal characteristics, social or interpersonal issues, or environmental considerations [18]. They can be defined as “those who seek information in a nonprofessional or informal capacity on behalf (or because) of others without necessarily being asked to do so” [15]. Proxy seekers may also be “experts,” such as health librarians or health care professionals, with the specialized knowledge or skills to use the information with the person with whom they share a personal relationship [19].

The role of proxy information seeking has been explored in the literature and has also been referred to as surrogate seeking or lay information mediation [12,20]. In one of the earliest models on information seeking behavior, Wilson [21] used pathways to explain different patterns of information seeking. In his model, the user encounters “information systems” that can be technology (eg, the internet) and mediators, and these systems connect the user to “information resources” or actual information. Of 10 pathways proposed in this model, 2 indicate seeking that is “conducted by a mediator to fulfill an information request” [21]. This phenomenon is also described in McKenzie’s [22] 2-dimensional model of information practices of women pregnant with twins. In her model, one of the modes of information practice is “by proxy,” where the person interacts with information through another agent, including “intermediaries or gatekeepers” such as friends or family members.

#### Social Support

Social support is one of the positive products of “social relationships” that may have short- and long-term effects on health, for better and for worse, depending on their quality and quantity [23]. A 2004 model by Uchino [24] describes 2 broad dimension of support: structure and function. Structural aspects of support are the extent or composition of one’s social network (size, contact, type, density, and strength) and the interconnections among them. Functions have 4 aspects that are highly related to each other: emotional, informational, tangible, and belonging. Most relevant to this review is informational support, which includes the provision of advice or guidance and may provide direction and carry an emotional message when received from a close source. Informational support could be construed as supportive, unsupportive, or mixed depending on the context [25-27].

Social support has consistently been linked to better health [24,28,29]. Several theories have been proposed to explain why this occurs; for example, social support can act as a mediator of stress that reduces its impact, thereby improving mental health [23]. Several studies have reported that those who perceive low social support experience increased stress and report a greater number of stressful events, while those who feel more satisfaction with their received social support report fewer emotional problems [30-33]. Another theory to explain the link between social support and better health is the provision of informational support, which encourages the receivers to manage their health. If we use pregnant women as an example, those who were more satisfied with perceived and received social support initiated prenatal care earlier than those who were less satisfied [34]. Pregnant women who received more informational support from people in their social network delivered babies with higher Apgar scores and higher birth weights [34,35].

#### Online Health Information Outcomes

**Figure 1:**
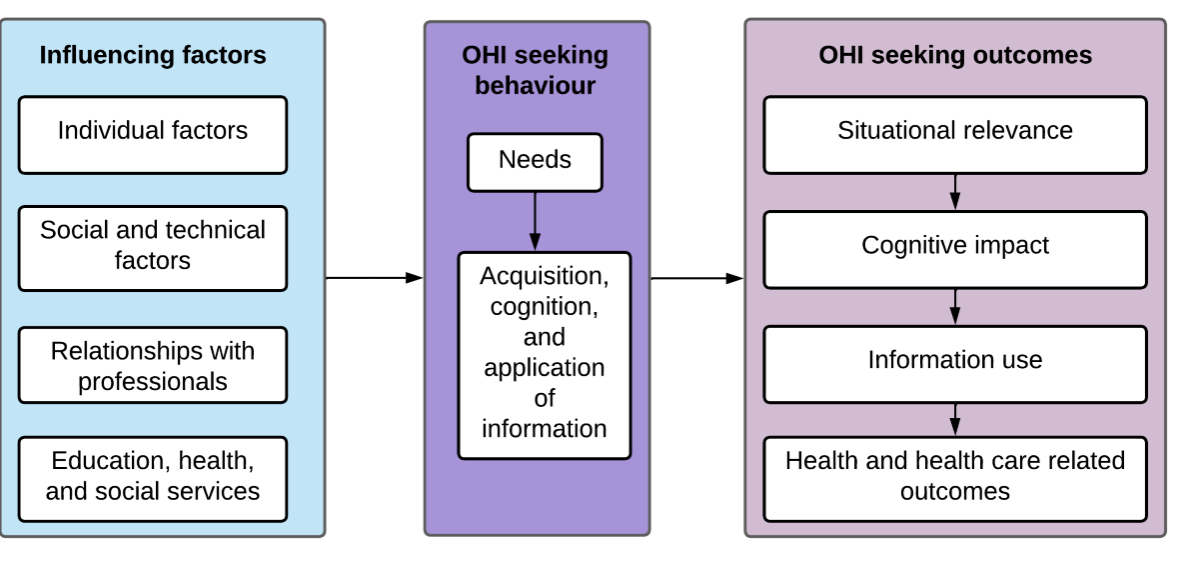
Online health information (OHI) outcomes conceptual framework.

A theoretical framework on OHI outcomes and the factors associated with these outcomes was developed by Pluye and colleagues [8] based on a systematic review with a framework synthesis. This framework was derived from previous research by the authors and combines the information theory and psychosocial theory of behavior. It includes 4 types of contextual factors that influence OHI outcomes: (1) individual factors (eg, health literacy); (2) social and technical factors (eg, access to the internet); (3) relationships with professionals (eg, satisfaction with health care provider); and (4) education, health, and social services (eg, access to a family doctor). It also includes 4 levels of individual outcomes of OHI seeking: (1) situational relevance, (2) cognitive/affective impact (eg, being able to understand the information or not liking the information found), (3) use (eg, in discussions with a health care provider or to make a medical decision), and (4) subsequent health/well-being outcomes of use (eg, improved health or reduced worrying) of information. These levels are presented in Figure 1. For each level, different types of outcomes were identified and validated using systematic mixed studies reviews and qualitative, quantitative, and mixed methods primary research studies [10,36,37]. 

However, this framework is focused exclusively on an individual perspective: it is the same person that starts the OHI seeking process and experiences the outcomes of this process. Studies that tested this framework therefore focused on people who used the OHI for their own health care and reported the health outcomes they themselves experienced. Little is known about what happens when the information need is to answer a question about someone else’s health or what is involved when the information is used with someone else (for providing social support) [14]. Therefore, to adapt this framework to the context of proxy OHI seeking, we are interested in 4 sections of this framework: (1) influencing factors of OHI seeking, (2) OHI seeking behavior including information needs, (3) OHI use, and (4) outcomes of OHI use.

#### Intersection of the 3 Concepts

There appears to be no comprehensive conceptual model on the outcomes of proxy OHI seekers using OHI to provide social support. Reifegerste et al [38] modified and extended the existing Comprehensive Model of Information Seeking (CMIS) with concepts of social network ties to predict proxy information seeking and the resulting social support intentions. They developed hypothetical scenarios (N=607) of people with varying severity in depression and with varying relationship closeness. Structural equation modeling was used to test the associations between the health-related factors (including demographics), proxy health information seeking intentions, and social support intentions. They hypothesized that support is the resulting action of proxy OHIS. This is an important study that modifies an existing information seeking model to proxy seeking; however, seeking and support were measured only as intentions. Moreover, the demographic characteristics were not found to be relevant, potentially due to the low variance of the study sample. Therefore, our review aims to build on this work by further exploring the context of proxy OHI seeking and the outcomes of using OHI to provide social support.

## Methods

### Design

A mixed studies review was conducted using a data-based convergent synthesis design in which qualitative and quantitative data were analyzed together using a qualitative thematic analysis [39,40]. A mixed studies review is ideal in this context because the evidence is from diverse fields of inquiry, and it uses diverse methods to provide a rich and highly practical understanding of complex health interventions [41,42]. Framework synthesis was then conducted to produce a revised conceptual framework.

### Eligibility Criteria

Table 1 lists the inclusion and exclusion criteria that were deemed appropriate for identifying relevant studies.

**Table 1:** Inclusion and exclusion criteria.

	Inclusion criteria	Exclusion criteria
Research methods	Primary and secondary research (ie, qualitative, quantitative, and mixed methods empirical studies and literature reviews)	Not empirical research or a literature review (eg, commentary, editorials, reports)
OHI^a^	Focus on online health information seeking Online resource about health and medical topics	No mention of OHI Offline health information resources (eg, books or pamphlets) Studies that tested specific online interventions (eg, testing the use of an e-kiosk or e-mental health services)
Proxy OHI seeking	Explore the phenomenon of proxy OHI seeking: Characteristics of proxy seekers Context of proxy OHI seeking Use of OHI Outcomes of OHI	No mention of proxy OHI seeking No mention of seekers that are physical members of the social circle that the person knows and is in contact with on a regular or semi-regular basis (eg, anonymous social media or online forum members) Exclude parents of young children or surrogate decision-makers of incapacitated adults (eg, unconscious patients in an ICU^b^)

^a^OHI: online health information.

^b^ICU: intensive care unit.

### Sources and Search Strategy

Papers were searched in 5 databases (Medline, PsycInfo, CINAHL, LISA, and Scopus) from inception to May 25, 2021. A search strategy was compiled with the help of a health librarian and included 2 main concepts: OHI and proxy OHI seeking or social support. The term “surrogate seeking” was discovered after reviewing articles from the first 4 databases and was thus added to the Scopus search strategy. The sets were combined using Boolean operators depending on the database being searched, as presented in Table 2. The search was limited to English and French languages, with no limit on years. All the records were transferred to a reference manager software (EndNote x8) and duplicates were removed using the Bramer method [43]. After the selection stage, additional potentially relevant records were retrieved by tracking the citations (snowballing) of the selected documents.

**Table 2:** Search strategy.

Database	Date of latest search	Search terms	Records, n
Medline	May 20, 2021	*social support/ AND online.mp. AND “Health Information”.af.	82
		“informational support”.mp. AND online.mp. AND “Health Information”.af.	14
CINAHL	May 20, 2021	“online health information” AND “social support”	16
		“online health information” AND “informational support”	5
PsycInfo	May 20, 2021	*social support/ AND online.mp. AND “Health Information”.af.	141
		“informational support”.mp. AND online.mp. AND “Health Information”.af.	36
LISA	May 20, 2021	“proxy” AND “information seeking” AND “online health”	54
		“social support” AND “online health” AND Information	294
Scopus	May 20, 2021	“surrogate” or “proxy” AND “information seeking” AND “online health”	25
		mediator AND “online health information”	118

### Selection of Relevant Studies

The 775 records were then imported into DistillerSR, a web-based application for conducting systematic reviews for selection [44]. For each record, eligibility codes were assigned according to the criteria described in Table 1. For every included record, the corresponding full-text publications were retrieved. Subsequently, full texts were imported into DistillerSR again and coded using the same eligibility criteria. Included studies were then exported into NVivo (Version 12).

### Data Extraction and Synthesis of Included Studies

Characteristics of the included studies and results related to the role of social support in OHI seeking and outcomes were coded in NVivo. A deductive-inductive analytical approach was adopted for thematic analysis of the extracted evidence [45]. A coding manual was developed following the framework proposed by Pluye et al [8] that included (1) characteristics of proxy-OHI seekers, (2) context of proxy-OHI seeking, (3) use of OHI by proxy seekers, and (4) outcomes of OHI use for the seeker and recipient. The codes were then progressively clustered into major themes and subthemes.

### Framework Synthesis

The initial framework in Figure 1 was revised following the qualitative synthesis stage. An iterative collaborative process was adopted over a series of meetings. All major themes were placed into textboxes and added to the figure representing the initial framework. Alternative figures were proposed until consensus was reached among the authors. The framework was then reviewed by 2 peer reviewers and presented at 2 research meetings (1 local and 1 international), and the feedback received was used to produce the final framework.

## Results

### Characteristics of Included Studies

**Figure 2:**
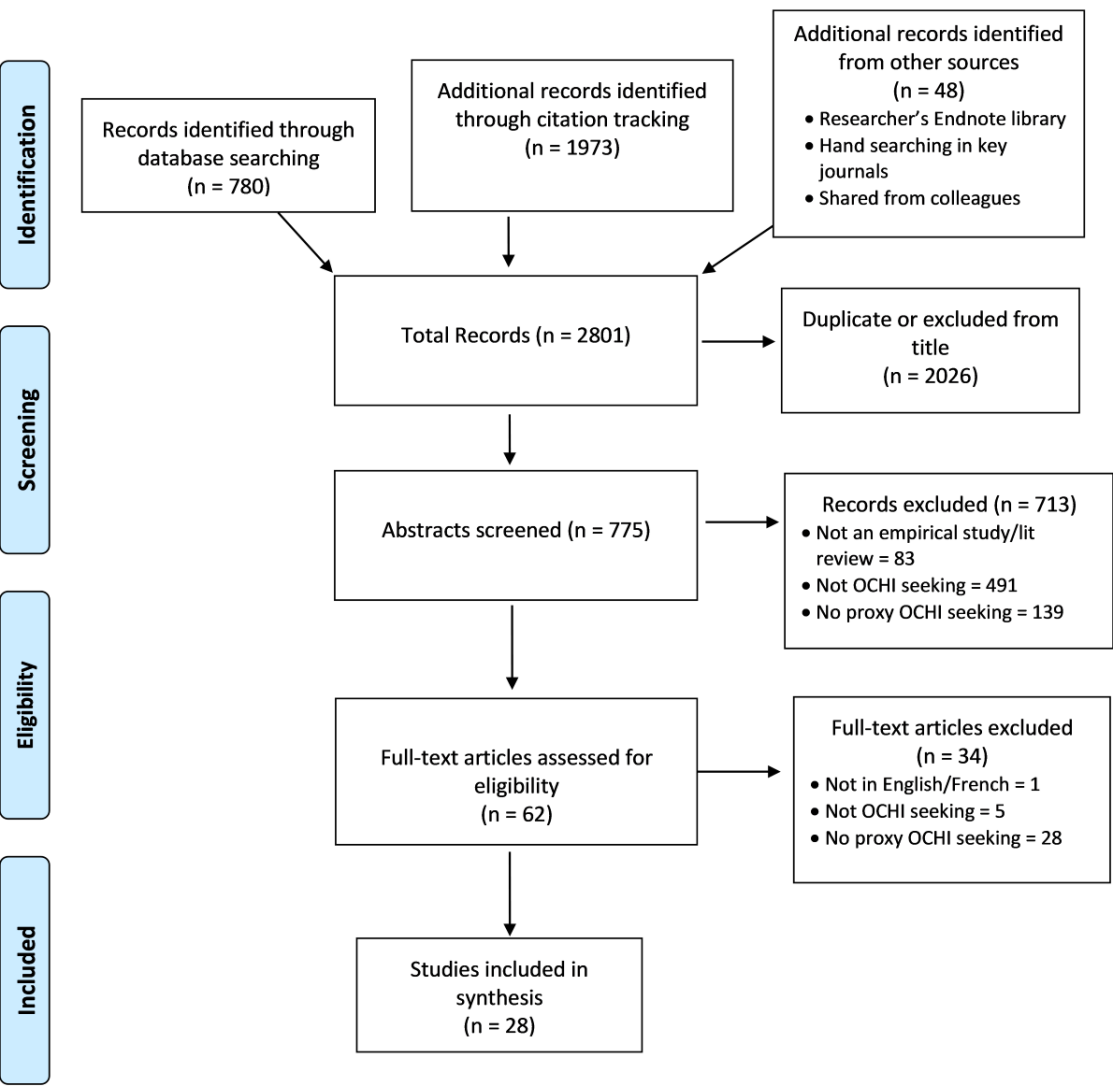
PRISMA (Preferred Reporting Items for Systematic Reviews and Meta-Analyses) flow diagram.

Of 775 unique records identified in our search, 28 were deemed relevant and included in our review (Figure 2). Those referred to 15 (53.6%) quantitative studies (including 1 experimental study), 10 (35.7%) qualitative studies, 1 (3.6%) mixed methods study, and 2 (7.1%) systematic reviews. Over half (n=16, 57.1%) of the empirical studies were conducted in North America. The corresponding 28 full-text articles were divided into 3 groups depending on who the focus of the study was: OHI proxy seekers (n=9, 32.1%), OHI recipients (n=2, 7.1%), or both (n=17, 60.7%). Full details of the study characteristics are in presented in Multimedia Appendix 1.

### Characteristics of Proxy Seekers

The results of a telephone survey of 18,750 European citizens show that 61% of those seeking OHI searched on behalf of someone else, and of those, 26.6% exclusively searched on behalf of someone else. These surrogate OHI seekers were more likely to live with others and more likely to search on behalf of their partners, children, or other family members rather than for friends or colleagues [11]. This finding was echoed in several studies that reported that the proxy seeker was most often a member of the same household or with whom the person had close ties [12,15,46-51].

This was especially highlighted in relationships where the proxy seeker considered themselves responsible for someone else’s health. We found 5 studies that focused on informal (unpaid) caregivers who reported higher and more constant proxy seeking behavior than noncaregivers [46,52,53]. A study exploring information seeking in families affected by multiple sclerosis describes the disease as a shared concern or responsibility that necessitates sharing information about it [54]. Dutta et al [55] described 3G households (3 generations of family members residing together) in Singapore, where the children and grandchildren play vital roles as sources of health information for grandparents.

Several other proxy seeker characteristics influenced OHI seeking behavior. One important factor is gender; 7 studies reported that most people who searched OHI on behalf of others were female [11,15,48,50,54,56,57]. Proxy seekers were generally younger and more educated [11,15,47,48,53,56,58] although 1 study reported that age, education, and income were not significant factors that influenced proxy OHI seeking behavior [59]. Another factor is related to the proxy seeker’s experience with OHI: respondents in several of the included studies were reported as having higher health literacy [12,54] and engaging in frequent OHI seeking behavior [11].

### Information Needs and Triggers of Proxy Seeking

OHI seeking was triggered by different reasons and at different times in the included studies (Table 3). The proxy seeker may be asked explicitly to search for OHI on behalf of someone who is unable to search for themselves, who has a complex health situation, or who needs to confirm information they had found online themselves [51,55,60,61]. On the other hand, more studies report that the proxy seeker initiates the search unsolicited out of interest [15,61], when they do not have enough information to support a person living with a health condition [47,54], immediately following a diagnosis [62-65], or following a visit with a health care provider [62,66]. Finally, the proxy seeker may also initiate the search themselves as a coping mechanism to help deal them with their emotions following the diagnosis of a loved one [53,61].

**Table 3:** Information needs and triggers of proxy seeking.

Code	Excerpt
Explicit request	“The carer may be asked to search for information on behalf of the person with cancer. This mostly occurs in situations where the patient does not have access to the internet or is not internet savvy or the person with cancer finds they are too ill to search.” [61]
To make a decision	“Both patients and caregivers also mentioned that they surfed the internet again at specific moments later during the lung cancer treatment trajectory, such as during chemotherapy, at the appearance of new symptoms or disease progression, or when having to make a choice between 2 treatment options.” [63]
To support someone with a health condition	“A high percentage of the 795 caregivers (87%) had used [the] internet to search for information about the disease of the patient they were taking care for in the last year prior to the survey.” [47]
Out of interest or obligation	“For Gina, a 26-year-old Chinese participant, her role as a granddaughter constitutes her interpretation of HIS^a^ as she mostly seeks out information for her grandparents. Jamila, a 37-year-old Malay woman, seeks out health information from the internet when one of her family members is not feeling well.” [55]
Following a health care practitioner visit	“Patients and caregivers mentioned that their need to seek information often arose once they had time to rest and think about what they had been told, often at a time when their questions could not directly be answered by the treating specialist anymore: ‘Once you have come home, you have forgotten half of what you have been told, which is exactly the moment you would want to ask something.’” [63]
Coping mechanism	“Carers also tended to act as ‘gatekeepers’ of information, and constantly sought new information as a means of coping.” [53]

^a^HIS: health information seeker.

### How Proxy Seekers Use OHI

Proxy seekers used OHI to better understand someone else’s illness or to help themselves feel more empowered in their role as caregivers [49,64,65,67]. Several studies reported the sharing of information between caregiver and patient either directly by sending them a link or printout or indirectly by discussing the information found [49,50,57,60,64,68]. One study describes sharing and resharing the information among a social network so that it reaches a larger number of people [55] or so that a larger number of people are involved in making sense of the information [57].

One aspect of providing informational support involves acting as gatekeeper and controlling incoming information flow for the person [15]. An included literature review exploring the role of caregivers of cancer patients identified this role in 3 included studies, potentially as a way to manage the cancer experience of the patient [61]. Families developed strategies for controlling information sharing, either explicitly with the patient or implicitly, especially if the information was potentially distressing or could lead to conflict [54,63].

Proxy seekers used the information in discussion with health care providers at a clinical visit [49,55,61,62,64]. This led to asking more questions and feeling more empowered during the visit, as well as involving the provider in the interpretation of the information [49,61,67]. In some cases, it led to requesting more testing or to trying a new treatment plan [62,69]. On the other hand, especially if the provider was not receptive to discussing the information, it also led to confronting or challenging the provider’s decision [62].

Proxy seekers also used the information to provide emotional [51,62] and material support, especially as informal caregivers [46,61] to the person. They used the information to change that person’s lifestyle; for example, mothers in 1 study cooked healthier food and encourage their families to walk together as a form of exercise [55]. In another study, the authors report that family members used the information to exert control on the patient, using techniques such as pushing or guilting [68].

### Outcomes of OHI Use

The outcomes reported by the included studies were overwhelmingly positive. Empowered by the informational they received, proxy seekers and effected individuals felt better informed and more confident, were able to discuss the information with their health care providers, and request different management options [61,62,69]. Information helped people make a health behavior change like quitting smoking [15]. It also helped lessen worries about their own health [15,66]. One study described a 87-year-old participant who reported she feels calmer when her grandchildren print out information and explain treatment options for her [55]. People described how having proxy seekers “care so much” about their health made them feel supported [51] and allowed them to have someone to talk to about their health [64].

Negative outcomes were rarely reported. A literature review found limited reports of patients’ anxiety or decisions to refuse cancer treatment [61]. There were 2 studies that reported that the proxy seekers themselves experienced more anxiety, sometimes because of information overload [65,66]. The proxy seeker and the person did not always have the same approach to OHI: in situations where the person did not want to “know” or ignored the information, this led to tensions and conflict [54,68].

### Revised Conceptual Framework

**Figure 3:**
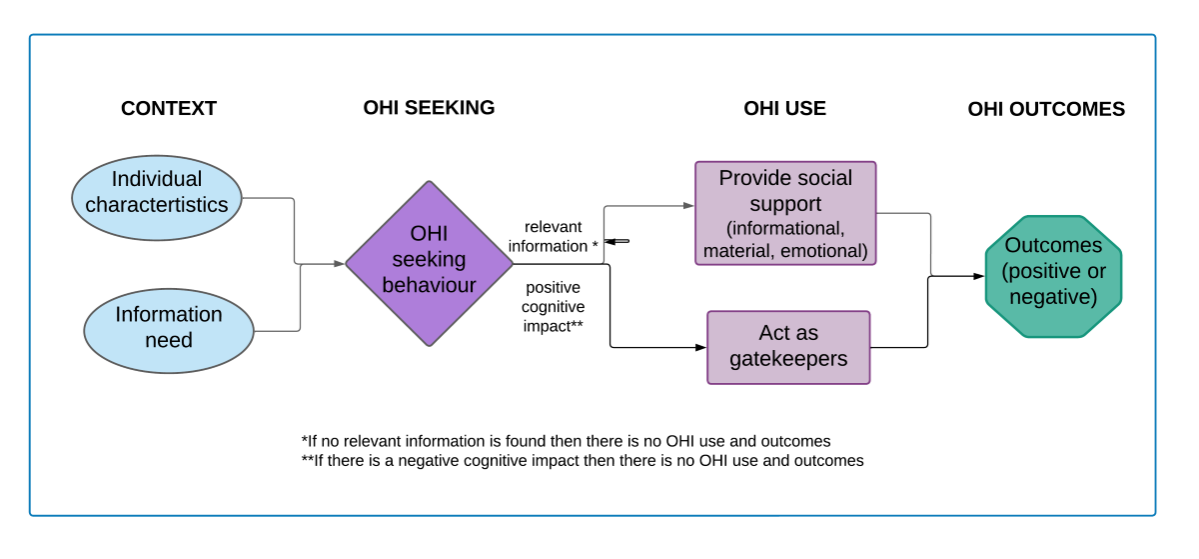
Outcomes of proxy online health information (OHI) seeking framework.

Figure 3 shows the revised conceptual framework after the review. The following paragraphs describe proxy seekers, their motivations, how they seek information, and their outcomes.

#### Who Proxy Seekers Are

Proxy seekers are more likely to be female and are also more likely to share health information with others, as they are considered the “central nodes” of health information within a community [70,71]. Moreover, they are more likely to be more educated, with higher eHealth literacy, and frequent internet users in general. Proxy seekers are likely to be in frequent contact with the people for whom they are seeking OHI and to report strong social ties with these people (eg, family members of the same household).

#### Why And When Does Proxy Seeking Occurs

The OHI seeking process is triggered by an explicit or implicit information need. Explicit information needs may be communicated to the proxy seeker with or without a request for informational support. Proxy seekers who are also informal caregivers may initiate OHI seeking as part of their caregiving responsibilities. The proxy seeker may also initiate the search themselves out of curiosity, for reassurance, or as a coping mechanism to help deal with their emotions following a diagnosis of their loved ones.

#### How Proxy Seekers Use Information

When proxy seekers find a situationally relevant information object that they understand or agree with (examples of positive cognitive impacts on the seeker), they can use it to provide social support for someone else. This support is most commonly informational: either by sharing the OHI found directly or discussing it with the person to help them make sense of it. Support may also be emotional or material, such as offering to cook meals. The proxy seeker also acts as an information gatekeeper by filtering the information for the person to reduce stress due to information overload.

#### Outcomes of OHI Use by Proxy Seekers

Using the information will lead to separate outcomes experienced by the person and the proxy seeker, which are generally positive; for example, feeling more confident discussing the information at a clinical visit. In situations where the information is conflicting or unsolicited, it may lead to negative outcomes such as increased worrying or worsening of an interpersonal relationship.

## Discussion

### Principal Results

To our knowledge, this is the first review to explore the outcomes of proxy OHI seeking and use of OHI to provide social support to others. We adapted a framework on individual OHI outcomes to proxy seekers and described and explained the context, use, and outcomes. Although there are 2 included reviews that reported interesting results, they did not fully address our question: the first explored the role of the internet in supporting and informing caregivers of people with cancer [61], and the second explored how informal caregivers of children with health care needs used internet-based health care services and resources [72]. Another relevant review that explored the proxy OHI seeking behavior of parents for their children and describing a conceptual model was not included in our review because parents are also proxy decision-makers for their children [73]. Another recent study adapted the existing Comprehensive Model of Information Seeking to surrogate health information seeking but did not explore the outcomes of social support [38].

### Comparison to Existing Models on OHI Seeking Outcomes

In his revised 1996 model, Wilson [74] added “information processing and use.” Our conceptual framework goes further and, in addition to describing the context of information seeking behavior by the proxy seeker, also explores OHI use and outcomes. Similar to the OHI outcomes framework by Pluye [8], our framework includes factors that influence information seeking behavior and leads to 4 levels of outcomes. The use of OHI in our framework revolves around types of social support, and the health and health care–related outcomes are reported by both the proxy seeker and the affected person. Moreover, we identified 2 additional consequences of informational support: sharing misleading information and acting as a gatekeeper to the information.

Our findings echo those of other studies exploring offline proxy health information seeking. In situations where the information need is explicit and the proxy seeker has high health literacy, informational support is associated with positive emotional support, and other outcomes are generally positive. First, people who can discuss the information they found with others are more likely to better understand the information, use that information to make decisions about their health care, and experience better health outcomes such as reduced worries [75-78]. Other potential outcomes include improvement in the receiver’s health, buffering of potential negative outcomes, and increase in perceived social support [9,32,79]. This is especially true if the provider has higher health literacy than the receiver, in that they are better able to explain, contextualize, or validate the information [80,81]. Some people may prefer information avoidance, defined as “any behavior designed to prevent or delay the acquisition of available but potentially unwanted information” [82], which may lead to tensions between the proxy seeker and the affected person.

Second, for the seekers themselves, these outcomes include a change in their relationship with the person (improved or worsened) and feeling more involved in the health care of others [83]. Moreover, social support providers who reported feeling more satisfied with their interaction with the person and who felt better about themselves after providing informational support were more likely to continue doing so and more likely to seek information from other sources [83]. Negative outcomes for the seekers reported include increased anxiety due to information overload. This is defined as “when the information processing demands on time…exceed the supply or capacity of time available for such processing” [84].

In situations where the informational support is unsolicited and the person does not feel that the information is relevant to their situation, interpersonal tensions may develop [14]. This may also occur in relation to sharing sensitive or intimate information with family members; for example, a study examining the effects of discussing information on sexuality and contraception on mother-daughter relationships reported that a strain in the relationship may develop [85]. In addition, sharing misleading health information from unreliable sources may also lead to negative health outcomes, as described in 2 recent systematic reviews [86,87]. More specifically, in this context, the seekers do not intend to cause harm and are in fact spreading misinformation that may lead to delayed care, decreased quality of life, and increased risk of mortality.

### Limitations

There are some limitations to our review. Unlike in a systematic review, only 1 reviewer carried out the selection phase, so some relevant studies may have been missed. However, our goal was to revise a framework and not necessarily to be exhaustive (in contrast to the needs of a systematic that aims to measure effectiveness of an intervention). Similar to other reviews, there may have been underreporting of negative outcomes due to publication bias. Finally, systematically reviewing all the models on information seeking behavior was beyond the scope of this review, but we reviewed and discussed the most common models with a specialized expert librarian.

### Directions For Future Research

Most studies on information seeking behavior do not explore how the information is used by proxy seekers, and what happens next [88]. While this review explores the outcomes of OHI proxy seeking, few studies report outcomes for the seekers themselves. As such, future empirical studies can focus on these outcomes from the seekers’ perspectives. Furthermore, little is known about which contextual factors or seeker characteristics are associated with positive and negative OHI outcomes. Future studies can test our framework in different contexts, revise it, and propose research-based solutions to help the proxy seekers use OHI with others.

### Conclusion

The outcomes of proxy OHI seeking constitute an important topic for both information specialists and health care practitioners. Members of a person’s social circle may help them overcome information-seeking barriers and illness challenges (eg, when they are too physically weak or mentally incapacitated to search themselves) [15]. People are sometimes more likely to turn to members of their social circle to make sense of OHI they find rather than discuss it with a health care professional [11]. By better understanding how affected people and their social circle use OHI together, OHI providers can better adapt their platforms and information to meet both their needs, and health care practitioners can target patients’ social circles with information for dissemination and use [16]. Potential public health intervention strategies can focus improving proxy OHI seeking and OHI use to promote positive outcomes for proxy seekers and the people they seek for through strategies that help proxy OHI seekers find relevant OHI, evaluate it, and use it appropriately. Strategies can also include extending social support networks for people without an effective social circle by identifying social support interventions from previous work that may be applicable in the context of proxy OHI seeking.

## Appendix

Multimedia Appendix 1 Characteristics of Included Studies.

## Abbreviations

CMIS: Comprehensive Model of Information Seeking
OECD: Organisation for Economic Cooperation and Development
OHI: online health information
