# Supplementary material for: Contexts and Outcomes of Proxy Online Health Information Seeking: Mixed Studies Review With Framework Synthesis
Source: J Med Internet Res. 2022 Jun 24;24(6):e34345. doi: 10.2196/34345 (PMC9270707; doi:10.2196/34345)
Supplement: Multimedia Appendix 1 [file jmir_v24i6e34345_app1.docx]

|  | **Country of study** | **Study Design** | **Study Objective** | **Participants** | **Entourage characteristics** | **OHIS behaviour** | **Reasons for OHIS or context** | **OCHI use** | **OCHI outcome** |
| --- | --- | --- | --- | --- | --- | --- | --- | --- | --- |
| **Proxy seekers and self-seekers** | | | | | | | | | |
| **Abrahamson 2008** | USA | MMR | Motivations for, barriers to, and effects of OCHI seeking and explored lay information mediary behavior (LIMB) characteristics in the consumer health information domain. | People seeking for information on behalf or because of someone else; & direct users: and & service providers | Female (77%, n=533), age 45–54  (33%, n514), and college graduates (30%, n513). |  | Out of interest or obligation.  Explicit request | LIMs share, store, or use health information that they determine is potentially useful; they also monitor information related to others’ needs and appear to assist others in processing information. | Affective: related to emotions (e.g., lessened worry about health care/procedures)  Cognitive: improved understanding of issues, terminology, etc.  Physical: led to a lifestyle or health behavior change, such as quitting smoking |
| **Bangerter 2019** | USA | Cross-sectional survey | Examine health information–seeking behaviors among caregivers and to identify caregiver characteristics that contribute to difficulty in seeking health information | Nationally representative sample drawn from the Health Information National Trends Survey (HINTS). Caregivers (n=391) | Caregivers that were non-white, less educated, privately insured, and without a regular health care provider reported lower confidence seeking health information. Caregivers with higher income reported more confidence seeking health information. |  |  |  |  |
| **Brown 2021** | USA | Qualitative | To investigate information behaviors in the context of health-related social control and the impact of control on patient health behavior | 38 family clusters with a total of 97 individuals. Eligible patients (Veterans Affairs Medical Center) were diagnosed with either Type 2 diabetes or HIV/AIDS and were willing to recruit family members involved in their care. | All families mentioned health-related social control- related behaviors in at least one interview contact. The most common form was pushing (telling patients what to do), questioning patient behavior, structuring the environment, and raising concerns. Guilting and repeating to reinforce points were the least commonly discussed. | Family gathered information individually or interactively, using methods including searching online, attending patients' healthcare appointments, and observing patients. | Family members acquired information to define problems and facilitate other information behavior that enacted social control | Information sharing took the form of persuasive social control strategies such as raising concerns and repeating points. When leveraging expertise, participants shared information from sources they deemed credible. Family often sought information individually when patients were first diagnosed “to set [their] mind [s] at ease….”, shared through two persuasive strategies, proxy informing and teaming up. | Ignoring was one response to norm enforcement; this involved simply refusing to listen to family directives or to take them into account, a form of passive resistance to control. |
| **Carpenter 2015** | USA | Cross-sectional survey | Describe the medication information-seeking behaviors of arthritis patients’ partners and explore whether partner medication information-seeking and information-sharing are associated with patient medication adherence | 87 patient–partner dyads: Eligible patients had osteoarthritis (OA) or rheumatoid arthritis (RA), were ~18 years old, could read English, had Internet access, and were currently taking ~1 medication. Patients’ partners were recruited by a snowball technique. | Partner information-seeking and information-sharing were positively associated (r = 0.51, p < 0.001). Partners sought more information about the patient’s arthritis medications if the patient reported a more complex medication regimen (r = 0.33, p = 0.002). Older partner age was associated with sharing more information with the patient (r = 0.25, p = 0.03). No other variables were associated with partner information-seeking or information sharing (supplementary materials). |  | Medication effectiveness was the topic partners discussed most with patients. A complex regimen may cause patients to explicitly ask their partners for treatment-related support, which may trigger partners to seek treatment-related information. | Most partners (97.7%) shared arthritis medication information with the patient; 15.1% shared rarely, 41.9% shared sometimes, and 40.7% shared often. | Neither partner information-seeking (r = 0.21, p = 0.06) nor partner information-sharing (r = 0.12, p = 0.31) were signiﬁcantly associated with patient medication adherence. |
| **Cutrona 2015** | USA | Cross-sectional survey | To identify those characteristics which differentiate surrogate seekers from those who seek information only for themselves. | Health Information National Trends Survey (HINTS) in 2011-2012. Among the 2,171 respondents who reported using the Internet to seek health or medical information in the previous 12 months, 66.6 % (n=1,461) had acted as a surrogate seeker. | 59.1 % of surrogate seekers were aged 35–64 compared to 49.7 % of self-seekers; p= 0.002). Those living with others were significantly more likely to report seeking information online for someone else Online surrogate seekers and self-seekers showed no significant differences in how they perceived their experience of information-seeking. Surrogate seekers and self-seekers were also equally confident that they could get advice or information about health or medical topics, if needed |  |  |  |  |
| **Cutrona 2016** | USA | Cross-sectional survey | To examine sociodemographic characteristics, health information seeking behaviors, and other online behaviors among health information brokers. | Health Information National Trends Survey (HINTS). Our final sample included 3142 respondents. Approximately half (54.8 %) of the respondents reported acting as health information brokers. | Brokers were more frequently female, reported higher incomes and higher educational levels. Respondents between the ages 35 and 64 acted as brokers most frequently. Compared to nonbrokers, a higher percentage of brokers were married. Compared to those with high school or less education, those with greater than high school education more frequently acted as brokers. |  |  |  |  |
| **Dolce 2011** | USA | Qualitative | To describe the experiences of cancer survivors and caregivers with healthcare providers in the context of the Internet as a source of health information. | Purposive sample of 488 cancer survivors, with varying cancer types and survivorship stages, and caregivers. |  |  | Several participants shared stories about not receiving the most up-to-date cancer information. | Collaborative healthcare providers were receptive to survivors and caregivers bringing knowledge and information gathered from the Internet to the clinical encounter. Participants exercised power through direct confrontation with their healthcare providers, which included behaviors such as questioning, persuasion, and coercion. Participants influenced their care and treatment plan by exerting persuasive power in their relationship with healthcare providers. |  |
| **Dutta 2018** | Singapore | Qualitative | Our study explores how meanings are assigned to HIS behavior: how do Singaporeans come to make sense of HIS? | Stratified snowball sampling strategy targeting participants (n = 100) that fit into a nationally representative demographic composition of the population wide census in Singapore. | In 3G (referred to as three generations of family members residing together) families, grandchildren play vital roles as sources of health information for grandparents, often themselves seeking out health information in response to a request from a grandparent. Living together across generations shapes the context of HIS and sharing. |  | When asked about their understanding of HIS, most participants referred to the roles they played within relationships, and the ways in which these roles necessitated HIS. E.g., granddaughter, family members, professional. “This is what we do in our culture. We have to take care of our grandparents”. | Mary, a 35-year-old Chinese woman, seeks out health information whenever her 72-year-old mother needs to know something. For Rani, a 55-year-old Indian woman, it is her husband who seeks out information from the internet and then educates her about various health-promoting habits. “I will pull out information from my mother’s health screening tests and discuss, I will also share this with my friends, and they will become part of the decision-making.” This back-and-forth process of information sharing serves as a framework for interpreting the information, and for collective sense making. | “She gets scared these days. At least, I can be there for her, and get the information on the treatment, the side effects. That calms her.”  “We will talk as a family about my health condition, and that makes me feel secure.” |
| **James 2007** | UK | Qualitative | To examine cancer patients’ and carers’ use of, and attitudes to, the Internet as an information source compared with other media | The study was set up in three Birmingham teaching hospitals: recruited 800 recently diagnosed patients, with any primary cancer, and 200 carers attending with a randomly chosen subset of the patients. | Signiﬁcantly more carers (48%) used the Internet compared with only 4.8% of patients. Carers were more likely to be the information seekers. Use of the Web among carers increased with level of education and also among those in ‘white collar’ as opposed to manual or skilled jobs. |  |  | Carers also tended to act as ‘gate keepers’ of information, and constantly sought new information as a means of coping. |  |
| **Mazanderani 2019** | UK | Qualitative | Explore the intra-familial dynamics of managing health information in the context of chronic illness | 77 interviews with people affected by Multiple Sclerosis in the UK (patients, partners, family members and close friends). | When one member of a couple (either the patient or their partner) avoided or ignored information, the other usually compensated by taking on the responsibility of managing it. Conversely, in cases where the patient was an avid information seeker, other family members tended to take a back seat, allowing the patient to take the lead. For many of our interviewees, it was a matter of personality and ‘natural’ abilities and inclinations, with the key information worker perceived as having better research, technical (especially Internet) or communication skills. |  | MS was seen as something that individuals within a relationship experienced differently, but faced ‘together’, and sharing a ‘life with MS’ often went hand-in-hand with the sharing information about it. | Our interviewees’ emphasis on sharing information (as well as on labouring through it) was balanced by an equally pronounced stress on controlling its ﬂow into, within and beyond the family unit, with families developing their own idiosyncratic strategies for doing so. In some cases, these strategies emerged spontaneously with little overt discussion, while in others they were the result of an explicit agreement. | Differences in approaches to health-related information could sometimes result in tensions and even conﬂict. This was especially notable in situations where one or more family member (commonly the patient) resisted receiving or sharing health-related information. |
| **Turner 2018** | USA | Lit Review | Report on the types of HI sources and information seeking practices used by older adults and the FF that support them. | 88 in-person interviews with older adults and 52 telephone interviews with FF | FF had an average age of 67.4 years, and were predominantly female (77%), white (87%) and had at least a bachelor’s degree (73%). 77% of FF reported using the internet on a daily basis. Several older adults mentioned that they placed a higher priority on HI from FF who were also healthcare professionals. | FF often sought input from other FF regarding HI. FF served as sounding boards for each other in thinking through HI for the older adult. | Older adults frequently chose to involve FF with their HI seeking. Older adults often used peers to “compare notes” about HI. Some older adults asked FF to find HI on their behalf. In addition, older adults frequently asked FF to suggest HI sources, and to help them make sense of HI. | FF frequently searched for health information for older adults.  “…I researched the drug that they wanted to put her on. And I helped her make up a list of questions that she wanted to ask when she went to her next appointment.” (FF14, daughter) FF often consulted with other FF to obtain and share information regarding the older adult’s health. |  |
| **Schook 2014** | Netherlands | Qualitative | To explore the reasons why lung cancer patients and caregivers search the Internet for information and ask online lung specialists questions on the DLIC’s interactive page, “Ask the Physician”, rather than consulting with their own specialist. | The sample comprised 5 lung cancer patients and 20 caregivers who posed a question on the interactive page of the DLIC website. |  | An element they mentioned with regard to the available information on the Internet was the difficulty of understanding or interpreting online information correctly, as they were lacking a doctor’s knowledge and felt overwhelmed by the vast amount of information given.  Patients and caregivers mentioned that sometimes they postponed or stopped their Internet search, for instance, because the information they encountered was too much. Not searching helped them to stay positive. | Both patients and caregivers also mentioned that they surfed the Internet again at specific moments later during the lung cancer treatment trajectory, such as during chemotherapy, at the appearance of new symptoms or disease progression, or when having to make a choice between two treatment options. Patients and caregivers mentioned that their need to seek information often arose once they had time to rest and think about what they had been told. | Both patients and caregivers also talked about the occurrence of tension when meeting their own information needs by searching the Internet. Specifically, caregivers realized that their needs were not always the same as the patients’ and experienced difficulties in dealing with the information they had collected. They felt torn by the dilemma of disclosing sensitive information or hiding it from the patients, as they wanted to protect them from (unwelcome) confrontations. For example, one caregiver said that he did not share the death of someone from his mailing group as he thought that this would be too much to handle for the patient. |  |
| **Nicholas 2003** | UK | Cross-sectional survey | To obtain information on the characteristics of the users of health information Web sites, to obtain feedback regarding for what they used online health sites and what were the perceived outcomes associated with using online health information. | A population of Internet users resident in the UK, yielding a sample of 1,322 respondents | Women were more likely to find information that helped someone else: 60% said they had done so compared with 53% of men This was also true of those respondents with children: 63% said this compared with 53%. Older respondents also were found to be more likely to find information that helped someone else. This was further true of those in a relationship. |  |  | A total of 58% said that information found enabled them to help someone else, while 51% said that it gave them information that the doctor had not given them. |  |
| **Oh 2015** | USA. | Cross-sectional survey | To investigate various factors predicting online health information seeking for themselves and online health information seeking for others in family caregivers to cancer survivors. | The data used in this study were taken from the Health Information National Trends Survey 4. A total of 1,113 family caregivers were included in this study. | Having surrogate OHIS was significantly correlated with lower age, being female, being married, being employed, better self-rated health, higher attention to the Internet, and higher trust in the Internet. |  |  |  |  |
| **Reifegerste 2017** | EU | Cross-sectional survey | RQ1: Does surrogate seeking also occurs in an offline context? RQ2: Are there differences between countries? RQ3: Are there differences between offline and online surrogate seekers and interactions of the information source type with social, demographic, and media-related characteristics? | Using a large-scale representative survey from the 28 member states of the European Union (N= 26,566), our data comprise all respondents who reported seeking health information online or oﬄine (n = 18,750; 70.6%). | The results of the multilevel model indicate that living together is  the most important predictors of surrogate health information seeking. People who lived with others were more likely to seek health information on behalf of someone else than those living in single-person households. In addition, being female or having a higher health status, higher health knowledge, and higher education were all positively associated with a higher likelihood of surrogate seeking. |  |  |  |  |
| **Reifegerste 2020** | Germany | Experimental | To apply and test the Comprehensive Model of Information Seeking to surrogate OHIS | The final sample comprised 607 German participants. | Direct experience was negatively related to OHIS intentions,  thereby indicating that those with more experience had lower intentions regarding surrogate OHIS. | Beliefs had a direct effect on utility; however, no direct effects were observed of demographic factors, salience, or experience on utility. Additionally, utility predicted surrogate OHIS intentions, and OHIS predicted social support intentions. | It should be also noted that the direct effects of the relationship between surrogate OHIS intentions and social support intentions, although statistically significant, were small. However, the direct effect of salience on support intentions was relatively strong. The support intentions seemed to go beyond the prerequisite of information seeking. |  |  |
| **Sadasivam 2012** | USA | Cross-sectional survey | To assess differences between self seekers versus those that act also as surrogate seekers. | Our analysis was conducted using data from the Pew Internet and American Life Project 2008 Health Survey. N=1250 information seekers who reported looking for health information online. Out of these, 56% (N = 705) reported looking for health information for others the last time they sought health information on the Internet. | In the bivariate analysis, gender, age, and education were not signiﬁcantly associated with surrogate seeking the last time the respondent went online Increasing household income was positively associated with surrogate seeking. Information seekers who reported being married and a parent were more likely to be surrogate seekers. Information seekers who self reported health status as good or excellent were more likely to be surrogate seekers. Information seekers having someone close to them with a medical or chronic health care problem were also more likely to be surrogate seekers Information seekers who reported being married/being a parent were more likely to be surrogate seekers. |  |  |  | Of the Internet health information seekers, 57% (N = 724) reported some impact of the health information. Out of these, 22% (N = 158) reported a major impact. There were no signiﬁcant differences in self-reported impact between the self seekers and surrogate seekers (P = 0.48). |
| **Proxy-seekers only** | | | | | | | | | |
| **Bouju 2014** | France | Cross-sectional survey | To determine the proportion of family members who carried out medical information Internet searches during the ﬁrst days of the ICU stay. | During the study period, 726 patients stayed in the ICU for over 48 h. The questionnaire was completed by 222 (36 %) visitors. | Before the sixth day, 45 % had used the Internet to search for medical information. Some patient and family member characteristics were associated with increased Internet use, including family age and education as well as patient length of stay |  |  |  | According to responders, the Internet seemed to have limited impact on the physician–family relationship, and only 49 % thought that Internet use was unavoidable. |
| **Chua 2020** | Singapore | Cross-sectional survey | To establish the prevalence of health-information-seeking behaviours among caregivers of cancer patient and their resource preference in order to guide practice. | Data were obtained via a self-reported questionnaire from caregivers of cancer patients at the National Cancer Centre Singapore. N= 986 | Compared with caregivers who have ever searched for cancer information, the non-searchers tended to be older, had primary and below qualifications. A high percentage of caregivers who have ever searched for cancer information were children taking care of their parents with cancer. | A high percentage (46%) of these caregivers was concerned about the quality of information they have found on the Internet. | A high percentage of the 795 caregivers (87%) had used Internet to search for information about the disease of the patient they were taking care for in the last year prior to the survey.  The top three topics being searched are treatment (35.6%), disease (35.6%) and side effects (26.5%) |  |  |
| **7 : Coder 2020** | USA | Cross-sectional survey | Explore the information needs and seeking behaviors of family members and friends who experienced a terminal cancer diagnosis of a loved one that included a predicted lifespan. | Respondents were parents (32%), children (23%), friends (5%), spouses/ partners (4%), and siblings (4%), with 32% representing other family members like grandparents and grandchildren |  | Among those participants who experienced problems, 31% (n = 12) felt  too overwhelmed/ anxious to research, 18% (n = 7) were unable to formulate question(s) and did not know which search terms/keywords to use, and 15% (n = 6) stated they did not have access to certain resources. | When asked if they wanted to immediately seek out medical information to understand the diagnosis, the majority answered Yes (72%, n = 54), followed by Maybe (13%, n = 10), No (7%, n = 5). | When asked how the medical information was used, to Better understand illness represented the largest response rate (33%, n = 55), followed by Cope with illness (25%, n = 43), Talk with physicians/other health-care providers (22%, n = 38), and to make medical decisions (17%, n = 29). Other (2%, n = 4) responses included researching optimal treatment plans and using the information to support the patient. | 47% (n = 28) of the participants stated that they did not experience information overload whereas 42% (n = 25) noted that they did experience information overload. Most respondents that reported information overload experienced negative effects: 48% (n = 24) experienced stress and anxiety, followed by 34% (n = 17) who could not absorb the information, and 16% (n = 8) quit the task at hand. |
| **Coffey 2017** | USA | Qualitative | To identify the preferred sources of health information for caregivers supporting individuals with injuries and to explore how access to this information could be improved. | 32 caregiver interviews, of which 16 providing care to a person with a TBI, 10 providing care to a person with a SCI, and 6 providing care to a person with a burn injury. |  | The majority of subjects (n=30) received injury-related information via the Internet through sites found using search engines (n=20) and medical websites (n=20). | Caregivers researched information that was directly related to supporting the individual receiving care. “Treatment” (n=14), “rehabilitation” (n=13), and “medication” (n=11) emerged as dominant themes, “long-term care” (n=15). | Caregivers reported instances of reliance on their own judgment based on independent research. This sometimes led to negotiation or collaboration with medical professionals. |  |
| **Kernisan 2010** | USA | Qualitative | To better understand what types of information are sought by those visiting a website focused on elder-care issues and to identify overarching themes that might inform future development of Internet resources related to caregiving and aging. | Data were obtained from Caring.com (a comprehensive resource for adults caring for aging parents). Of 2161 submitted surveys, 1467 of 1838 free-text comments (80%) were included in the content analysis. | Those caring for parents were more likely to be female. |  | Many respondents indicated that they were looking for specific advice on the practical aspects of managing the daily living needs of another person, with a majority of these making reference to caring for parents with dementia or other frailty. |  |  |
| **Kinnane Milne 2010** | Various | Lit Review | To review the best available  evidence for how carers use the Internet for cancer-related information and support. | Articles in English concerning family, carer, friends of cancer patient’s use of the Internet were identified. |  |  | The carer may be asked to search for information on behalf of the person with cancer (where the patient does not have access to the Internet or is not Internet savvy or the person with cancer finds they are too ill to search). Acting out of concern or kindness, the carer may initiate the search themselves and provide the patient with details of what they have found. | Of note, although carers may access the Internet for information for the patient, they can also act as ‘gatekeepers’ and may not pass on certain information. The information found is used in a variety of ways: to inform treatment-related decisions, to check up on medical decisions and to increase knowledge. Other uses included confirming existing treatments and treatment-related decisions for the patient as the best possible options. | Reading Internet information obtained by carers can result in a small percentage of patients requesting tests or treatment. Rarely does reading information found on the net result in patient decisions to refuse or stop cancer treatments. Reading the information can lead to increased confidence, being better informed and being able to discuss the information with a health care professional. There were limited reports of anxiety or confusion after reading information found on the net. |
| **Kirschning 2007** | Germany | Cross-sectional survey | Why do the family members conduct research on the net? Do they use the net for themselves or do they pass on Internet-based information to the patients? How does the information passed on influence the therapy decisions? | It was focused on the internet use of family members of women with breast cancer and men with prostate cancer (n=113). | More than half of the respondents were between 40 years and 60 years old. Significantly more than half of the respondents were employed; only one out of five women questioned was  a housewife. The respondents lived overwhelmingly in a partnership, somewhat more than half lived in a family. Half of the respondents had a household income of over €2,500/month. The women respondents were overwhelmingly daughters, daughters-in-law and friends of women with breast cancer. The men questioned were overwhelmingly partners of women with breast cancer. | Most of the respondents printed out the important information (86%). Half set up a collection (50%). Each fifth respondent mailed or e-mailed the information to the patient. | The results relate to a spectrum of  people who had experiences with all disease phases. Often, they researched for a patient who was not familiar with the medium (60%). | The family members used the Internet primarily to inform themselves (91%) but also to convey information to the sick person (78%). Half of the respondents integrated Internet information into a medical consultation (56%). Significantly more than half produced a printout of the information they wanted to talk about (62%). Those respondents tried actively to involve the physicians in the interpretation of the information. | The detectable reactions were surprising. Fewer than half noticed an interested reaction from the physician (41%).These differing aspects can clarify why, with a third of  the respondents, there was the predominating impression that the physician was overwhelmed (36%). |
| **Li 2015** | USA | Cross-sectional survey | Describes the online caregiving information that caregivers searched for and identifies factors related to their search. | 800 informal caregivers for community-residing older adults aged 65 and over. This study is a secondary data analysis of the Caregiving in the U.S. 2009 survey | The majority of caregivers were White (60.4%) and female (65.5%). Three-quarters of the caregivers (74.3%) had some college education, and two-thirds (68.0%) were employed at some point during the time they provided caregiving. Nearly four in 10 (40.0%) had annual household incomes less than $50,000. The main contribution of this study is its focus on the relationship between caregivers’ use of the Internet and their information and service needs and caregiving contextual factors. |  | The information that interested the searchers most included care receivers’ conditions or treatments (77.2%), available services for care receivers (52.7%), and care facilities (35.3%). Nearly 11% of caregivers looked for information about support for themselves. This study shows that caregivers who reported higher levels of information/ service needs are likely to conduct more frequent Internet searches. |  |  |
| **Tonsaker 2017** | Canada | Qualitative | This study investigated how caregivers access and use information on the Internet about caregiving and their perspectives on the design and features of a new personal health experiences (PHEx) website. | Three focus groups of caregivers for a total of 16 participants in a university-affiliated hospital in Quebec. |  | They begin their search using a general information portal where they type in a question or keyword and choose amongst websites, they compare content and also incorporate external influences, including personal background and preferences, as well as suggestions from family and friends. |  | Participants in the present study experienced empowerment by enhancing interactions with doctors or other health care professionals, through managing their own health, their care recipient’s health, and their role as a caregiver, as well as by developing social connections and receiving support online. |  |
| **Self-seekers only** | | | | | | | | | |
| **Simon-Schramm 2008** | USA | Cross-sectional survey | This study considers cancer-related Internet use among families and friends of cancer patients, and how that use of the Internet may affect patients and patient care | The questionnaire was administered to 120 patients who were treated in a cancer center in Ohio, USA. | In 73% of all cases, patients reported receiving some online information from their social networks. This online information was received from a spouse or partner (n = 33; 38%), an older child (n = 14; 16%), another relative (n = 10; 12%), a friend (n = 9; 10%), or other individuals (e.g. parents, siblings, and, co-workers; n = 21; 24%) who helped access, provide, and/or interpret online information | Of the 86 patients who reported receiving personal  Internet-use support, most (n = 63; 73%) did not ask their personal caregivers to provide this support. Instead, caregivers took the initiative in accessing the Internet for information to share with patients. |  | Online information obtained by personal caregivers was typically shared with patients telephonically and/or through face-to-face contact (n = 77; 89%), or through computer printouts (n = 55; 63%). Only 23 (26%) patients reported receiving emailed cancer-related information from their personal caregivers. Less than one third (n = 35; 29%) of all patients reported having talked to their health care providers about their use, or their family’s use, of the Internet. | Of the 36 patients asked this question, roughly half (n = 19; 53%) reported that the information was ‘‘very helpful,’’ more than one third (n = 13; 36%) felt that the information was ‘‘somewhat helpful,’’ and three (8%) believed the information was ‘‘not too helpful.’’ Several patients commented that their caregivers, rather than they themselves, found it useful and informative to go online. It helped stimulate and give structure to interactions with family and friends. |
| **Song 2019** | China | Qualitative | To explore the factors that influence the old people to adopt proxy internet health information seeking (PIHIS) | 20 old people in rural areas of Xuzhou City, Jiangsu Province who have experience in proxy health information seeking |  |  | “I am lack of the knowledge and skills to use computers and smartphones. If I need to search health information online, I can only ask my children to help me” | “My daughter often searches some information on the Internet and tells me how to pay attention to my body and what food I can’t eat. I’m glad to see that my children care so much about me” | “My daughter often searches some information on the Internet and tells me how to pay attention to my body and what food I can’t eat. I’m glad to see that my children care so much about me” |
